# *Acinetobacter* pneumonia: Is the outcome different from the pneumonias caused by other agents

**DOI:** 10.4103/1817-1737.62472

**Published:** 2010

**Authors:** Ebru Cakir Edis, Osman N. Hatipoglu, Ozlem Tansel, Necdet Sut

**Affiliations:** *Department of Pulmonary Medicine, Trakya University Medical Faculty, Edirne, Turkey*; 1 *Department of Infectious Diseases and Clinical Bacteriology, Trakya University Medical Faculty, Edirne, Turkey*; 2 *Department of Biostatistics, Trakya University Medical Faculty, Edirne, Turkey*

**Keywords:** *Acinetobacter* spp., hospital-acquired pneumonia, risk factors, survival, therapeutic success

## Abstract

**BACKGROUND:**

The principal aim of the present study was to determine whether *Acinetobacter* spp. pneumonia differs from hospital-acquired pneumonias (HAPs) caused by other agents with respect to therapeutic success and survival rate.

**METHODS:**

This study includes 140 adult patients diagnosed with HAPs caused by identified etiologic agents between March 2005 and February 2006. These patients were divided into two groups according to the agent responsible for their infection (*Acinetobacter* spp. [*n* = 63] or non-*Acinetobacter* spp. [*n* = 77]). The groups were compared in terms of risk factors, therapeutic success and six-week survival rates.

**RESULTS:**

Previous antibiotic use and the risk of aspiration were independent factors responsible for the development of *Acinetobacter* spp. pneumonia. Hypoalbuminemia, steroid use and the use of a mechanical ventilator were determined to be mortality-associated independent risk factors for *Acinetobacter* spp. pneumonia. The clinical success rate at the end of therapy was 41.6% and, at the sixth week, the survival rate was 35% among patients in whom *Acinetobacter* spp. was the causative agent. Conversely, in the control group, these values were 43 and 32%, respectively (*P* > 0.05). We found that the use of the appropriate antibiotics for the treatment of *Acinetobacter* spp. pneumonia was an important factor in survival (*P* < 0.001).

**CONCLUSION:**

The outcomes of *Acinetobacter* spp. pneumonia do not differ from HAPs associated with non-*Acinetobacter* spp. in terms of therapeutic success and survival rates.

*Acinetobacter* species are Gram-negative, nonfermentative, nonspore-forming, nonmotile, aerobic coccobacillary organisms. The prevalence of infections caused by *Acinetobacter* spp. has increased rapidly since the 1970s.[1]

*Acinetobacter* spp. are frequently encountered agents responsible for hospital-acquired pneumonia (HAP), especially the late-onset, ventilator-associated pneumonias (VAPs). *Acinetobacter* spp. rapidly acquire antibiotic-resistance mechanisms, which may contribute to its virulance. Hospital-acquired pneumonias caused by *Acinetobacter* spp. lead to significant mortality and morbidity because of both their intrinsic and acquired resistance.[2] However, there have been a limited number of studies comparing *Acinetobacter* spp. pneumonia and non-*Acinetobacter* spp. pneumonia.[3,4]

The primary aim of the present study was to determine whether *Acinetobacter* spp. pneumonia is different from HAPs caused by other agents in terms of therapeutic success and survival rate. The secondary aim was to assess the drug resistance characteristics of *Acinetobacter* spp. and the independent risk factors associated with the development and mortality of pneumonia caused by *Acinetobacter* spp. in our hospital.

## Methods

This study included 140 adult patients with HAP of known etiologic agent, admitted to the Trakya University Medical Faculty Hospital between March 2005 and February 2006. When the etiologic agent of their pneumonia could be identified, the patients were divided into two groups: those with HAP caused by an *Acinetobacter* spp. [*n* = 63] and those with HAP caused by a non-*Acinetobacter* spp. [*n* = 77; control group].

### Definitions

- Hospital-acquired pneumonia: Hospital-acquired pneumonia was defined according to the standard definitions of the American Thoracic Society guidelines for the management of adults with hospital-acquired pneumonia.[5]
- Ventilator-associated pneumonia: Pneumonia that developed 48 h after being connected to a ventilator was accepted as VAP.[5]
- HAP developed in patients undergoing immunosuppressive therapy: HAP developed in patients undergoing immunosuppressive therapy for solid organ tumors, hematologic malignancy, or rheumatoid disease. The use of >20 mg corticosteroid for at least 3 weeks was assumed the required criteria for being considered as immunosuppressive therapy.
- Pneumonias that developed >4 days after hospitalization were considered late-onset pneumonias.[5]
- Patients were considered to have severe HAP in the presence of one of the following criteria:[6]
  - arterial oxygen pressure (PaO_2_)/fractioned oxygen percentage (FiO_2_) < 250;
  - severe sepsis or septic shock findings; or
  - bilateral or multilobar involvement, cavitations, abscess, effusion and rapid progression.
- Multidrug-resistance (MDR) was defined as resistance to more than one of the following five drug classes: antipseudomonal cephalosporins, antipseudomonal carbapenems, β-lactam/β-lactamase inhibitor combinations, antipseudomonal fluoroquinolones and aminoglycosides.[7]
- Clinical success was marked by a decline or disappearance of symptoms (fever, cough, sputum, and dyspnea) in the patients receiving antibiotic therapy.[8]
- Appropriate antibiotherapy: The patients were given at least three days of antibiotics appropriate to the antibiogram of the identified etiologic agent.

### Study protocol

Data obtained from the patients included in the study were collected prospectively. The approval of the local Ethical Committee was obtained during the planning phase of the study and each patient (or his/her caregivers) gave informed consent prior to participation in the study. The patients' demographic data, risk factors and the severity and the day of onset of the pneumonia were recorded. Chest X-rays, blood count, biochemical parameters, arterial blood gases and CRP were obtained from each patient prior to the initiation of therapy. Blood and sputum/tracheal aspirate cultures (if possible) were also obtained. Sputum/tracheal aspirate specimens containing >25 leukocytes per field and <10 epithelial cells per field were deemed acceptable for culture. Pleural fluid samples were obtained from patients with previously detected pleural effusions. Computed thoracic tomography was performed as required.

The decisions pertaining to the diagnosis and treatment of HAP were managed multidisciplinarily by the clinician responsible for the care of the patient, the pulmonary disease specialist, and the infectious disease specialist. Clinically recovered patients were discharged after posttreatment evaluation. Discharged patients were called for follow-up at the sixth week.

### Statistical methods

Descriptive statistics and frequency analyses were calculated for the different types of cases. Kaplan-Meier methods were used for the survival analysis. Univariate Cox regression analysis was used to assess factors that might independently affect mortality. After the univariate analysis, variables with *P* < 0.1 were analysed using a multivariate Cox regression model.

Univariate logistic regression analysis was used to examine independent risk factors on the outcome (*Acinetobacter* vs. non-*Acinetobacter*). Multivariate logistic regression analysis with a backward stepwise method was used to examine significant factors (*P* < 0.10) obtained from a univariate model.

A *P* value of <0.05 was considered to be statistically significant. Statistical analyses were conducted using SPSS 9.0 (SPSS Inc., Chicago, IL, USA) statistical software.

## Results

Of the 63 patients in whom *Acinetobacter* spp. were isolated, 38 (60%) were male and 25 (40%) were female. The mean age was 64.43 ± 13.89 years, with a range of 35 to 95 years. Thirty-eight of these patients were hospitalized in the internal medicine service, whereas 25 patients were hospitalized in the surgery service. The clinical services from which *Acinetobacter* spp. were most frequently grown were neurology (*n* = 29, 46%) and brain surgery (*n* = 7, 11.1%). In patients in whom *Acinetobacter* spp. were isolated, 43 had HAP, 17 had VAP and 3 had pneumonia that developed during immunosuppressive therapy. In the group in which etiologic agents other than *Acinetobacter* spp. were isolated, 45 had HAP, 17 had VAP and 15 had pneumonia that developed during immunosuppressive therapy (*P* = 0.027).

Seventy *Acinetobacter* spp. infections were isolated from the 63 patients followed during the course of the study. Of these, 49 were isolated from tracheal aspirates, 5 from blood and tracheal aspirates, 1 from pleural fluid and 15 from blood cultures (in which other possible infection sources, such as central venous catheter, etc. had been excluded). Three different *Acinetobacter* spp. strains were isolated from 2 patients, whereas 2 different *Acinetobacter* spp. strains were isolated from 3 patients. When the demographic characteristics and risk factors of the patients were compared between the groups from which *Acinetobacter* spp. and other causative agents were isolated, the risk factors with a *P-* value of < 0.1 according to univariate analysis were examined by multivariate analysis [Table 1]. Previous antibiotic use (*P* = 0.02) and the risk for aspiration (*P* = 0.02) were determined to be significant risk factors in the group from which *Acinetobacter* spp. were isolated. Previous antibiotic use and aspiration risk increased the risk for *Acinetobacter* spp. infection nearly three-fold (95% CI, 1.15–7.32) and nearly 2.5-fold (95% CI, 1.10–5.51), respectively [Table 2]. (Aspiration risk: Patients with confusion due to any cause, especially neurological diseases and patients lying supinely.)

**Table 1:** Demographic variables and risk factors in HAP patients with *Acinetobacter* spp. and with non-*Acinetobacter* spp.

Variables factors	*Acinetobacter* spp. n (%)	Non-*Acinetobacter* spp. n (%)	*P*
Age (Mean ± SD)	64.43 ± 13.89	62.36 ± 16.9	0.43
Gender (M)	38 (60.3)	47 (61)	0.93
Late-onset pneumonia	55 (87.3)	66 (85.7)	0.78
Day of pneumonia	14.75 ± 16.07	17.39 ± 17.55	0.36
(Mean ± SD)			
Severe pneumonia	48 (76.2)	51 (66.2)	0.20
Patient risk factors			
COPD	5 (7.9)	5 (6.5)	0.74
Heart failure	16 (25.4)	20 (26)	0.93
Diabetes	14 (22.2)	9 (11.7)	0.09
CRF	12 (19)	13 (16.9)	0.74
CVD	38 (60.3)	33 (42.9)	0.04
Malignancy	9 (14.3)	22 (28.6)	0.04
Antibiotic use	55 (87.3)	49 (63.6)	<0.01
Hypoalbuminemia	57 (90.5)	66 (85.7)	0.39
Smoking	23 (36.5)	29 (37.7)	0.88
Alcohol intake	4 (6.3)	7 (9.1)	0.55
Risk for aspiration	50 (79.4)	42 (54.9)	<0.01
Risk factors due to medical interventions			
Use of H2 blockers	33 (52.4)	38 (49.4)	0.72
Steroid use	29 (46)	36 (46.8)	0.93
Use of cytostatics	2 (3.2)	13 (16.9)	0.01
Use of sedatives	6 (9.5)	4 (5.2)	0.32
Risk factors due to invasive interventions			
Previous operation	14 (22.2)	13 (16.9)	0.42
Emergent intubation	19 (30.2)	16 (20.8)	0.20
CPR	4 (6.3)	5 (6.5)	0.97
Being connected to MV	17 (27)	17 (22.1)	0.50
Urinary catheter	51 (81)	52 (67.5)	0.07
TPN	15 (23.8)	11 (14.3)	0.15
Central catheter	23 (36.5)	21 (27.3)	0.24
Nasogastric tube	45 (71.4)	39 (50.6)	0.01
Tracheostomy	15 (23.8)	7 (9.1)	0.02

COPD = Chronic obstructive pulmonary disease, CRF = Chronic renal failure, CVD = Cerebrovascular disease, CPR = Cardiopulmonary resuscitation, MV = Mechanical ventilator, TPN= Total parenteral nutrition, SD = Standard deviation

**Table 2:** Evaluation of risk factors via multivariate analysis found to be significant by univariate analysis

Variables	Univariate analysis			Multivariate analysis		
		
	*P*	HR	%95 CI	*P*	HR	95% CI
Diabetes	0.090	2.15	0.86–5.38	0.070	0.40	0.15–1.08
CVD	0.040	2.02	1.03–3.98	0.950	1.02	0.39–2.68
Malignancy	0.040	2.40	1.01–5.68	0.780	0.85	0.27–2.68
Antibiotic use	<0.01	2.92	1.63–9.42	0.020	2.91	1.15–7.32
Aspiration risk	<0.01	3.20	1.50–6.83	0.020	2.47	1.10–5.51
Use of cytostatics	0.010	6.19	1.34–28.59	0.160	3.18	0.62–16.23
Urinary catheter	0.070	2.04	0.92–4.49	0.260	0.51	0.15–1.65
Nasogastric tube	0.010	2.43	1.20–4.93	0.520	1.35	0.52–3.45
Tracheostomy	0.021	3.12	1.18–8.23	0.090	2.40	0.86–6.70

HR = Hazard ratio, CI = Confidence interval, CVD = Cerebrovascular disease

Determination of *Acinetobacter* strains is based on drug susceptibility patterns. When antibiotic susceptibility was examined, the highest susceptibility was to netilmicin [Table 3]. Sixty-four of the 70 isolated *Acinetobacter* spp. strains were multidrug resistant. Since tigecycline and colistin were not available in our country during this study, sensitivity for these two drugs was not investigated.

**Table 3:** Antibiotic susceptibilities of *Acinetobacter* spp. strains

Antibiotics	%
Netilmicin	93
Cefepime	69
Piperacillin–tazobactam	65
Ceftazidime	50
Ampicillin–sulbactam	48
Imipenem	39
Meropenem	36
Amikacin	34
Cefoperazone	23

Clinical success after treatment was achieved in 26 patients (41.3%) from whom *Acinetobacter* spp. were isolated, but this rate was reduced to 22 patients (34.9%) after the follow-up (sixth week). In the group from which an agent other than *Acinetobacter* spp. was isolated, clinical success rates were 43 and 32%, respectively, and no significant difference was determined between these rates (*P* = 0.8).

**Figure 1:**
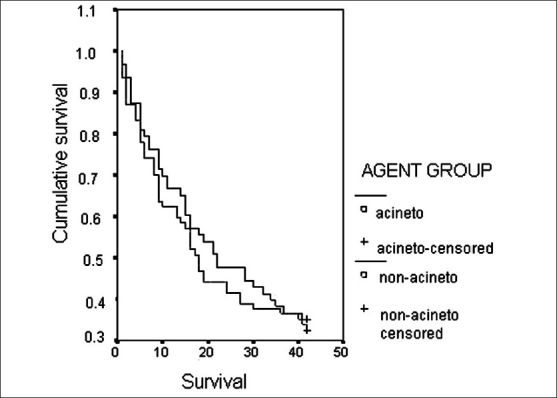
Survival analysis of *Acinetobacter* spp. and non-*Acinetobacter* spp. groups

Forty-one of 63 patients (65%) died during the six-week period. According to the Kaplan-Meier survival analysis, the survival rates at the 3^rd^, 7^th^, 14^th^ and 42^nd^ days were 87, 76, 65 and 35%, respectively. No significant difference was determined in terms of survival rates between the group in which *Acinetobacter* spp. were isolated and the groups in which agents other than *Acinetobacter* spp. were isolated [Figure 1]. When *Acinetobacter* spp. strains were evaluated for sensitivity and resistance to imipenem, no difference was demonstrated in terms of survival in patients with pneumonia in whom non-*Acinetobacter* spp. were grown (*P* = 0.77).

In the *Acinetobacter* spp. group, only *Acinetobacter* spp. were isolated from 34 patients, and in 29 patients, the isolated agents were polymicrobial. In the control group, a single agent was isolated in 62 patients, while polymicrobial agents were isolated from 15 patients. In neither group were the effects on mortality dependent upon the causative agent(s) being single or polymicrobial.

**Figure 2:**
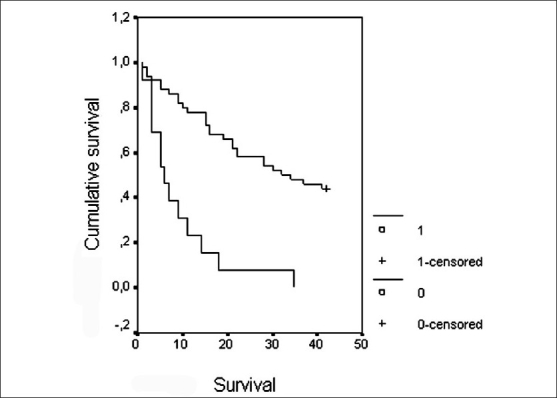
The effect on survival of taking an appropriate antibiotherapy in patients with *Acinetobacter* spp. pneumonia

In both the *Acinetobacter* and non-*Acinetobacter* spp. groups, there were no significant differences among the patients who were exitus before receiving the appropriate antibiotics (*P* = 0.57). It was found that the appropriate antibiotic treatment for *Acinetobacter* spp. was significant in the survival rate in the *Acinetobacter* spp. group (*P* < 0.001), while it was nearly significant in the non-*Acinetobacter* spp. group (*P* = 0.054). However, among the patients who were appropriately treated with antibiotics in both groups, no significant differences in terms of survival were found (*P* = 0.20). For the *Acinetobacter* spp. group, mortality increased 5.01 (95% CI, 2.47–10.18) times when the patient did not receive the appropriate antibiotics [Figure 2].

The impact of the variables on the six-week survival in patients with *Acinetobacter* spp. pneumonia was analysed by univariate Cox regression analysis [Table 1]. Variables with a *P*-value of < 0.1, according to the univariate analysis, were evaluated with multivariate analysis. Hypoalbuminemia (*P* = 0.04), steroid use (*P* = 0.002), and the use of a mechanical ventilator (*P* = 0.036) were determined as factors that independently affected survival. The risk of mortality was increased 3.24-fold by hypoalbuminemia (95% CI, 1.05–9.96), 3.07-fold by steroid use (95% CI, 1.49–6.34) and 2.19-fold by the use of a mechanical ventilator (95% CI, 1.05–4.58; Table 4). The effect of *Acinetobacter* spp. bacteriemia on mortality was not found.

**Table 4:** Evaluation of risk factors via multivariate analysis found to be significant by univariate analysis which impacted mortality

Variables	Univariate analysis			Multivariate analysis		
		
	*P*	HR	95% CI	*P*	HR	95% CI
Severe pneumonia	0.05	2.19	0.97–4.97	0.43	1.48	0.55–3.99
CRF	0.07	1.95	0.94–4.04	0.08	1.99	0.91–4.35
Hypoalbuminemia (<3.5 mg/dl)	0.08	2.26	0.88–5.81	0.04	3.24	1.05–9.96
Risk for aspiration	0.04	2.65	1.03–6.81	0.87	1.09	0.36–3.24
Use of H2 blockers	0.09	1.71	0.91–3.19	0.80	1.09	0.52–2.31
Use of steroids	<0.01	3.11	1.64–5.90	<0.01	3.07	1.49–6.34
Use of sedatives	0.02	2.86	1.11–7.36	0.25	1.92	0.62–5.95
MV	0.01	2.34	1.22–4.50	0.03	2.19	1.05–4.58

HR = Hazard ratio, CI = Confidence interval, CRF = Chronic renal failure, MV = Mechanical ventilator

## Discussion

This prospective observational study investigated 140 patients with a diagnosis of hospital-acquired pneumonia (HAP). We intended to determine whether *Acinetobacter* spp. pneumonia is different from HAP caused by other agents with regard to therapeutic success and survival rate. We found that previous antibiotic use and the risk of aspiration were independent predictors of the development of *Acinetobacter* pneumonia, but we did not find differences in the clinical success or in the six-week survival rates.

We found only two studies comparing *Acinetobacter* spp. pneumonia and non-*Acinetobacter* spp. pneumonia in English literature.[3,4] However, all studies were performed in intensive care units (ICUs) on intubated patients. The difference between the present study and the other studies is that the present study not only included all HAPs developed within the entire hospital, but also investigated the patients with HAP, VAP and pneumonia occurring during immunosuppressive treatment.

Risk factors for *Acinetobacter* spp. pneumonia were defined as neurologic problems and aspiration, previous antibiotic use and being connected to a ventilator.[2,9–12] In the present study, aspiration increased the risk for *Acinetobacter* spp. pneumonia nearly 3-fold, and previous antibiotic use increased the risk for *Acinetobacter* spp. pneumonia nearly 2.5-fold. Similar to our study, another study which evaluated VAPs that grew *Acinetobacter* spp. (*n* = 41) versus non-*Acinetobacter* spp. (*n* = 40) found that previous antibiotic use was determined to be a risk factor for ventilator-associated *Acinetobacter* spp. pneumonia, according to multivariate analysis.[3] In another study, which analysed 46 VAPs associated with *Acinetobacter* spp. and 79 VAPs associated with other pathogens, previous ceftriaxone and ciprofloxacin use were determined to be significant risk factors.[4]

*Acinetobacter* spp. pneumonia was recently identified as an important cause of mortality, particularly among patients who acquire pneumonia in ICUs. We found the high proportion of *Acinetobacter* spp. pneumonia among HAP in our institution to be very significant. In 63 of the 140 patients with HAP included in the study, *Acinetobacter* spp. were the responsible agents. This situation shows that, in our hospital, there are problems with regard to infection control measures and antibiotic use.

Infections caused by MDR *Acinetobacter* spp. are difficult to treat and are associated with high mortality. Carbapenems are frequently used in such patients; however, resistance develops rapidly.[13] The *Acinetobacter* spp. strains grown in the present study were susceptible to netilmicin (93%) and cefepime (69%). The susceptibility rate to imipenem was 39%. MDR *Acinetobacter* spp. strains were grown in 64 (91%) patients. In a study previously performed in our hospital, the susceptibility of imipenem in *Acinetobacter* spp. strains between 1994 and 1995 was 100%, which was then reduced to 35% between 2003 and 2004.[14] In Turkey, in a study performed on VAPs caused by *Acinetobacter* spp. strains, resistance to ceftazidime, imipenem and ciprofloxacin was determined to be 60, 64 and 80%, respectively, and the most susceptible antibiotic was cefoperazone-sulbactam.[15] Since tigecycline and colistin were not available in our country at the time of this study, sensitivities for these two drugs were not investigated. When it is considered that most *Acinetobacter* spp. cases are resistant to most drugs, the use of these antibiotics could affect the results of *Acinetobacter* spp. pneumonia treatment.

In patients in whom *Acinetobacter* spp. was isolated, the clinical success rate after treatment was 41%, which was reduced to 35% after follow-up. No significant difference was shown between the groups in which *Acinetobacter* spp. and non-*Acinetobacter* spp. were isolated in terms of clinical success, after both treatment and follow-up. Although the number of patients in whom pneumonia developed while under immunosuppressive therapy was significantly higher in the non-*Acinetobacter* spp. agent group, no significant difference was found between the two groups in terms of survival rates. There was also no significant difference in terms of survival rates between the patients with pneumonia caused by imipenem-susceptible or -resistant *Acinetobacter* spp. strains and the patients with non-*Acinetobacter* spp. In a study performed in patients with VAP, survival was evaluated between groups in which *Acinetobacter* spp. (imipenem-susceptible - imipenem-resistant) and non-*Acinetobacter* spp. were isolated. Similar to the present study, no difference was found between the two groups in terms of survival.[3,16,17] In the present study, we also found that receiving the appropriate antibiotics is a factor affecting survival in the *Acinetobacter* spp. group.

When the mortality-associated risk factors were evaluated, it was shown that hypoalbuminemia, steroid use and the use of a mechanical ventilator increased mortality. Although there are studies investigating the risk factors affecting in-hospital mortality in ventilator-associated pneumonias caused by *Acinetobacter* spp., no study evaluating mortality-associated risk factors in patients with only HAP and with *Acinetobacter* spp. growth exists in the literature.[3,18]

In conclusion, *Acinetobacter* spp. pneumonia does not differ from HAPs caused by non-*Acinetobacter* spp. agents in terms of therapeutic success and survival rate. Patients with HAPs caused by *Acinetobacter* spp. have a high risk of aspiration, the incidence of which is gradually increasing in patients who have previously received antibiotic therapy.
